# FAIDR-Desi: interactive software for feature analysis and design of intrinsically disordered regions

**DOI:** 10.1093/bioinformatics/btag715

**Published:** 2026-09-24

**Authors:** Tian Hao Huang, Robert Strome, Julie D Forman-Kay, Alan M Moses

**Affiliations:** Department of Biochemistry, University of Toronto, Toronto, ON, Canada; Department of Molecular Medicine, SickKids, Toronto, ON, Canada; Department of Cell and Systems Biology, University of Toronto, Toronto, ON, Canada; Department of Cell and Systems Biology, University of Toronto, Toronto, ON, Canada; Department of Biochemistry, University of Toronto, Toronto, ON, Canada; Department of Molecular Medicine, SickKids, Toronto, ON, Canada; Department of Cell and Systems Biology, University of Toronto, Toronto, ON, Canada

## Abstract

**Summary:**

FAIDR-Desi is a non-technical user interface for design of intrinsically disordered regions (IDRs) based on greedy optimization of sequence features (simple sequence statistics correlated with IDR function, including amino acid composition, residue patterning and short linear interaction motifs), using more features (127) than other IDR design approaches. FAIDR-Desi can be used to design synthetic IDR mimics to establish feature sufficiency for function, or to knock out features to show necessity for function, exemplified with experiments on the yeast Cox15 mitochondrial targeting signal.

**Availability and implementation:**

Software is freely available on the web at https://moses-lab.github.io/faidr_desi/ and an archival version of the source code is available on Zenodo (https://doi.org/10.5281/zenodo.22036189).

## 1 Introduction

Standard protein bioinformatics approaches based on 3D structure and positional conservation of residues are relatively uninformative about the sequence-function mapping for intrinsically disordered regions (IDRs), which are found in about 60% of the human proteome (Tsang *et al.* 2020) and regulate key biological processes (Van Der Lee *et al.* 2014, Holehouse and Kragelund 2024). Aberrant IDRs are involved in many diseases, including cancers and neurodegenerative pathologies (Uversky *et al.* 2008, Uversky *et al.* 2014, Tsang *et al.* 2020). Previously, we developed a bioinformatics approach enabling functional insights based on IDR bulk sequence features (Zarin *et al.* 2019, 2021) including composition (McConnell and Parker 2023), short linear interaction motifs (Garabedian *et al.* 2021, Lu *et al.* 2022), and residue patterning statistics (Cohan *et al.* 2021, Ruff *et al.* 2026). We showed that these IDR sequence features are evolutionarily conserved (Zarin *et al.* 2019, Pritišanac *et al.* 2024), and demonstrated that specific sequence features are sufficient for function by designing synthetic IDRs that preserve certain sequence features (Strome *et al.* 2023). More recently, similar approaches have been developed to interrogate IDR function based on sequence features (Cohan *et al.* 2021, Garabedian *et al.* 2021, McConnell and Parker 2023, Ruff *et al.* 2026) and to design IDRs having particular properties (Sherry *et al.* 2017, Garabedian *et al.* 2021, Emenecker *et al.* 2023, Kilgore *et al.* 2024).

However, the software tools for designing IDR sequences to fit sequence features or ensemble properties (Emenecker *et al.* 2023, Pesce *et al.* 2023, Kilgore *et al.* 2024, Krueger *et al.* 2026, Tesei *et al.* 2025, Novak *et al.* 2026) have typically been limited to an audience with assumed experience in python and package installation. Colab notebooks have been a reasonable compromise for many non-technical users but often rely on installing large python dependency chains on each run (i.e. at runtime), which leads to computationally redundant project builds on every use of the tool and potential for breaking changes in any dependency to affect the existing deployment. Here we introduce FAIDR-Desi (*F*eature *A*nalysis of *I*ntrinsically *D*isordered *R*egions *Desi*gn Tool), a web-based IDR design software tool hosted at https://moses-lab.github.io/faidr_desi/. FAIDR-Desi provides a runtime-dependency-free graphical user interface (GUI) that allows users to easily compute sequence features and design novel IDR sequences for experiments to demonstrate the sufficiency and necessity of a curated set of sequence features.

## 2 Functionality

FAIDR-Desi has three functionalities:

### 2.1 Showing the sufficiency of sequence features with feature mimics

Users can design a novel synthetic IDR sequence whose features match or “mimic” a target sequence’s features (referred to as a “feature mimic” IDR). The curated features include bulk properties such as charge and hydrophobicity, residue and repeat content and matches to short motifs from ELM (Dinkel *et al.* 2014, Pritišanac *et al.* 2024). To match the features of an arbitrary IDR, FAIDR-Desi uses a brute-force greedy algorithm starting from a random initial protein sequence (Strome *et al.* 2023). For each iteration, it selects its next point mutation by going through all possible point mutations, computing the normalized sequence feature vector (each entry is a *Z* score compared to the feature mean and variance of all human or yeast IDRs), computing the Euclidean distance of the mutated sequence’s feature vector to the target feature vector in this normalized feature vector space, and then choosing the mutant sequence with the lowest Euclidean distance to the target. This process is repeated until the program does not find any point mutations lowering the sequence feature distance.

Feature mimic IDRs are useful for IDR design because IDRs with similar sequence features have been shown to have similar biological functions (Zarin *et al.* 2019, 2021, Pritišanac *et al.* 2024, Ruff *et al.* 2026). Thus, users can design novel synthetic sequences with low positional sequence identity/similarity to natural sequences but are nonetheless expected to mimic the function of the input IDR. These designs can be used to test sufficiency of the measured IDR features.

### 2.2 Showing the necessity of sequence features with feature knockouts

The software can design a synthetic IDR sequence that ablates a set of user-chosen sequence features while preserving all (or most) other sequence features. FAIDR-Desi’s ablation algorithm uses a similar greedy feature optimization algorithm as described above but starts with the input sequence and calculates the distance away from a “knock out” target feature vector. The “knock out” target feature vector is the same as the input sequence’s features except the features to be knocked out have been set to be the minimum possible value over all human or yeast IDRs. To evaluate the extent to which the selected features have been successfully knocked out, the software also shows a side-by-side comparison of the reference sequence’s features and those of the designed knock out.

Feature knockout IDRs are useful in feature-function hypothesis tests because they attempt to account for confounding effects of knocking down unintended features. As an example, in a naïve mutational knock out approach, motifs to be knocked down may be replaced with alanine, but ablating too many motifs with this approach could significantly increase the alanine content of the IDR, introducing a confounding factor. FAIDR-Desi therefore provides the feature knockout functionality to generate less confounded IDRs for these types of experiments.

### 2.3 Computing our curated set of sequence features

For general analysis of IDR sequences, the software can compute the sequence features of a given sequence and report them as *Z*-scores compared to the mean and variance of all human or yeast IDRs.

## 3 Example usage

We experimentally demonstrate an example usage of our software in Fig. 1D, starting with a wild-type yeast IDR, the Cox15 N-terminal IDR (depicted in cyan). This IDR is known to be responsible for the mitochondrial localization of Cox15. In previous work (Strome *et al.* 2023), we designed a feature mimic IDR (depicted in pink) using FAIDR-Desi. This feature mimic IDR preserves many of the sequence features of our wild-type Cox15 IDR, as demonstrated by the mimic and wild-type IDRs having a net charge of +8 and a charge patterning parameter *κ* (Das and Pappu 2013) of −0.56. By co-expressing a feature mimic IDR-containing construct with GFP and the wild-type Cox15 tagged with mScarlet, images showed that the synthetic IDR construct colocalizes with the wild-type Cox15 IDR. This supports the hypothesis that IDR features are sufficient to recapitulate IDR function in this case.

**Figure 1:**
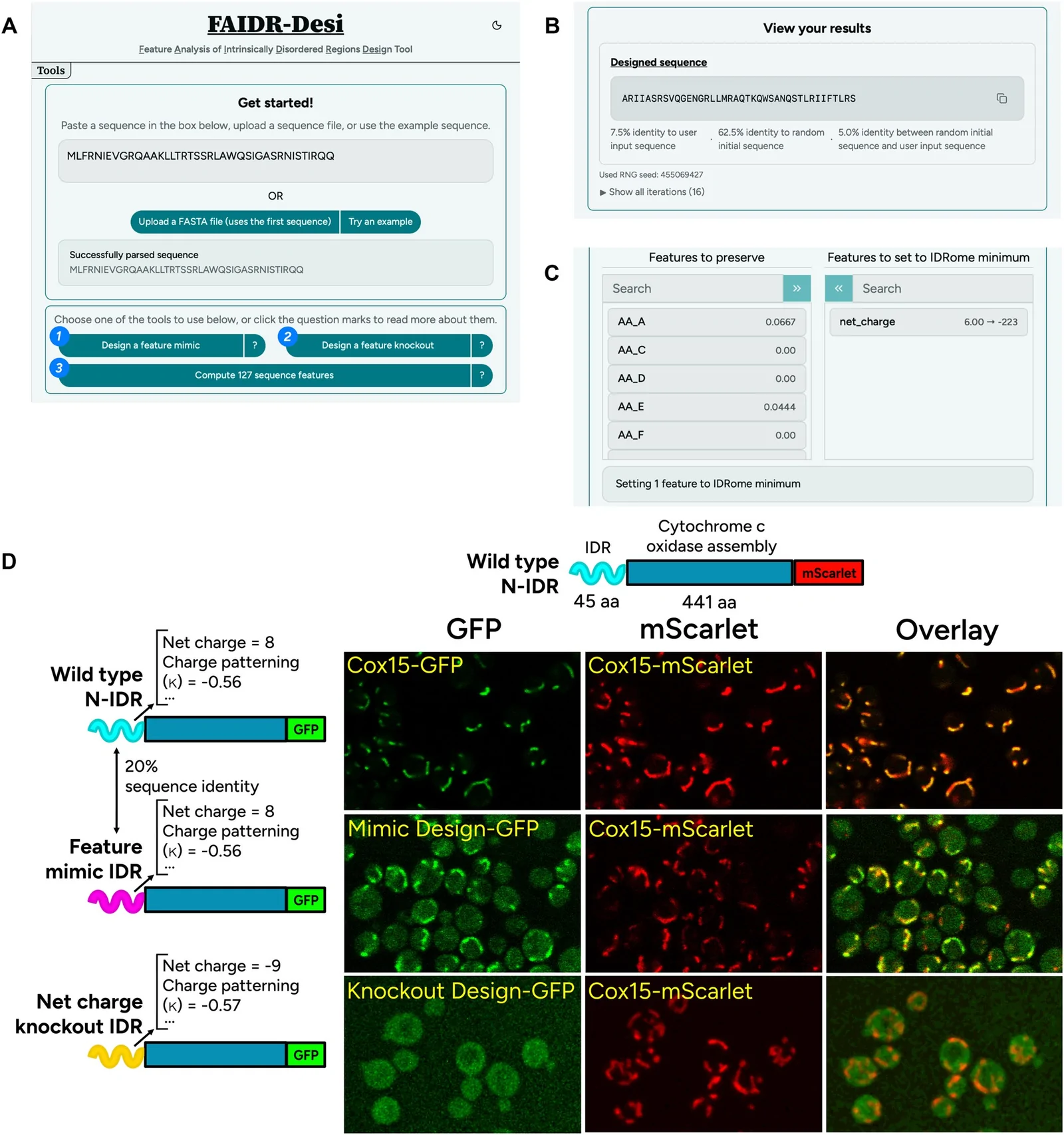
FAIDR-Desi, a simple IDR design GUI. (A) Main page, including three buttons labelled in this image with light blue circles which lead to sections (A.1) for generating a synthetic sequence that greedily matches features of an input sequence, (A.2) for knocking out specific features in the input sequence while preserving other features, and (A.3) for computing features of the input sequence. (B) Example of a designed sequence using the “feature mimic” functionality, along with sequences for and information about each design iteration in an expandable section below. (C) Panel in the “feature knockout” section, where users can choose which features to knock out by clicking features on the left list and then clicking the ≫ button to move them to the list on the right. (D) Experimental demonstration of feature sufficiency and feature necessity using FAIDR-Desi designs of the mitochondrial targeting signal of yeast protein Cox15. The net charge and charge patterning of the sequence features are shown beside the IDRs demonstrating that: our sequence features are preserved between the feature mimic and wild-type despite their low sequence similarity, and net charge is lowered from 8 to −9 while preserving other features like charge patterning in the net charge knockout. Microscopy images for three BY4741-derived strains stably co-expressing wild-type Cox15 tagged with mScarlet (as depicted on top) and a Cox15 IDR variant (wild-type/feature mimic/net charge knockout), which was concatenated to Cox15Δ1-45 (depicted as a dark blue rectangle) tagged with GFP.

We next wanted to find necessary features of Cox15’s N-terminal IDR. Previous work has demonstrated the importance of positive charges (Fukasawa *et al.* 2015, Garg and Gould 2016, Zarin *et al.* 2021), so we used FAIDR-Desi to design a “net charge knockout” IDR (depicted in yellow) by starting with the feature mimic IDR and setting net charge as low as possible (Fig. S1, available as supplementary data at *Bioinformatics* online). This net charge knock out IDR has a low net charge of −9 while preserving many of the other features, as demonstrated by having a charge patterning of −0.57, which is very close to the starting charge patterning value. Using the same experimental methods as previously described (Strome *et al.* 2023), Fig. 1D shows that a construct containing this knockout IDR failed to colocalize with the wild-type IDR, supporting the necessity of net charge in this IDR’s function.

## 4 Implementation details

FAIDR-Desi is a GUI that is written in React/TS and whose IDR design implementation is written in Rust and compiles to web assembly, run on the user’s browser. This architecture enables IDR design code that is: (1) easy for non-technical users to use as they don’t need to install anything and can merely visit the webpage to use the software, (2) standalone, since the web assembly file includes all dependencies statically without the need for runtime dependency installations, and (3) runnable with no need for an internet connection (other than the initial downloading of the app), because of the use of web assembly which runs in nearly any modern browser. The source code is available at https://github.com/moses-lab/faidr_desi and on Zenodo (https://doi.org/10.5281/zenodo.22036189).

## 5 Limitations

One limitation of our software is that it is hardcoded to use a set of 127 sequence features that have been introduced in previous work (Strome *et al.* 2023). Our features were manually curated and will not satisfy the requirements for all experiments. For more advanced users, our associated Colab notebook (https://colab.research.google.com/drive/1LiIZgeUwig VrKcFHib_huXezUTUR446u) enables custom feature design.

Another limitation comes from the time complexity of our simple brute-force point mutation checking algorithm, which is O(N^4^). The number of sequences checked by our algorithm scales as O(N^2^), coming from the fact that the number of mutations per each iteration is O(N), and the number of iterations is also O(N) if one assumes that in the typical case some expected fraction of the initial random sequence must be modified at least once. The time complexity to compute the feature vector for each checked sequence is also O(N^2^), as there exists an O(N^2^) feature in our feature set (SCD, a measure of charge patterning). On an Apple M1 chip in Safari, FAIDR-Desi designs a feature mimic of length 150 residues in 20 s, one of length 450 residues in 4 m, and one of length 1000 residues in 38 m. This simple slow algorithm has been sufficient for our design needs but raises challenges for the current software being used to generate synthetic IDR libraries or design for very long IDRs. Existing algorithms in the literature, such as Markov Chain Monte Carlo (MCMC) sampling (Pesce *et al.* 2023) or generating multiple mutations at once (Emenecker *et al.* 2023), could be used to make our IDR design software faster.

Another key limitation is due to non-independence between our IDR features. Attempting to knock out one such sequence feature while keeping a highly correlated feature preserved may result in the design algorithm not fully ablating the chosen feature. An illustrative example is shown in Fig. S2, available as supplementary data at *Bioinformatics* online where the user is trying to knockout the fraction of charged residues (fcr) of a sequence. The user attempts to knock out charged residues by setting the following features to zero: fcr, basic residues, acidic residues, the percentages of Asp/Glu/Lys/Arg, and Asp/Glu/Lys/Arg repeats. However, the user has not selected to modify several features which depend heavily on having charged residues, such as chain expanding residues (fcr + prolines) and a charge clustering measure (kappa). Running our program on the IDR encompassing residues 750–900 in yeast protein Mcm3 (P24279), it fails to reduce the charged residues below 20% (which is about 32 charged residues). Charges that are part of a charge cluster are not removed by our design algorithm. Users of the feature knock out tool are able to compare the features calculated for the reference and designed sequences using the table provided to ensure that the desired knockout-feature has been sufficiently knocked out.

## 6 Conclusions

Sequence feature-based IDR design has been utilized to uncover sufficient and necessary features responsible for IDR functions. Current software tools (Emenecker *et al.* 2023, Pesce *et al.* 2023, Kilgore *et al.* 2024, Krueger *et al.* 2026, Tesei *et al.* 2025, Novak *et al.* 2026) for feature-based IDR design are found primarily as python libraries or Colab notebooks, which have their limitations. FAIDR-Desi is, to our knowledge, the first software for feature-based IDR design that requires no runtime/manual dependency installation processes while simultaneously bypassing the need for a maintained server by utilizing user hardware to run computations. This enables highly accessible and intuitive feature-based IDR design for non-technical users with essentially no dependencies or potential for breaking changes. Ultimately, it is hoped that this functionality will stimulate significant design efforts by the biological community to explore new IDR biology and underlying mechanisms.

## Supplementary Material

btag715_Supplementary_Data
