# Supplementary material for: FAIDR-Desi: interactive software for feature analysis and design of intrinsically disordered regions
Source: Bioinformatics. 2026 Sep 24;42(10):btag715. doi: 10.1093/bioinformatics/btag715 (PMC13654200; doi:10.1093/bioinformatics/btag715)
Supplement: Supplementary file 1 [file btag715_supplementary_data.pdf]

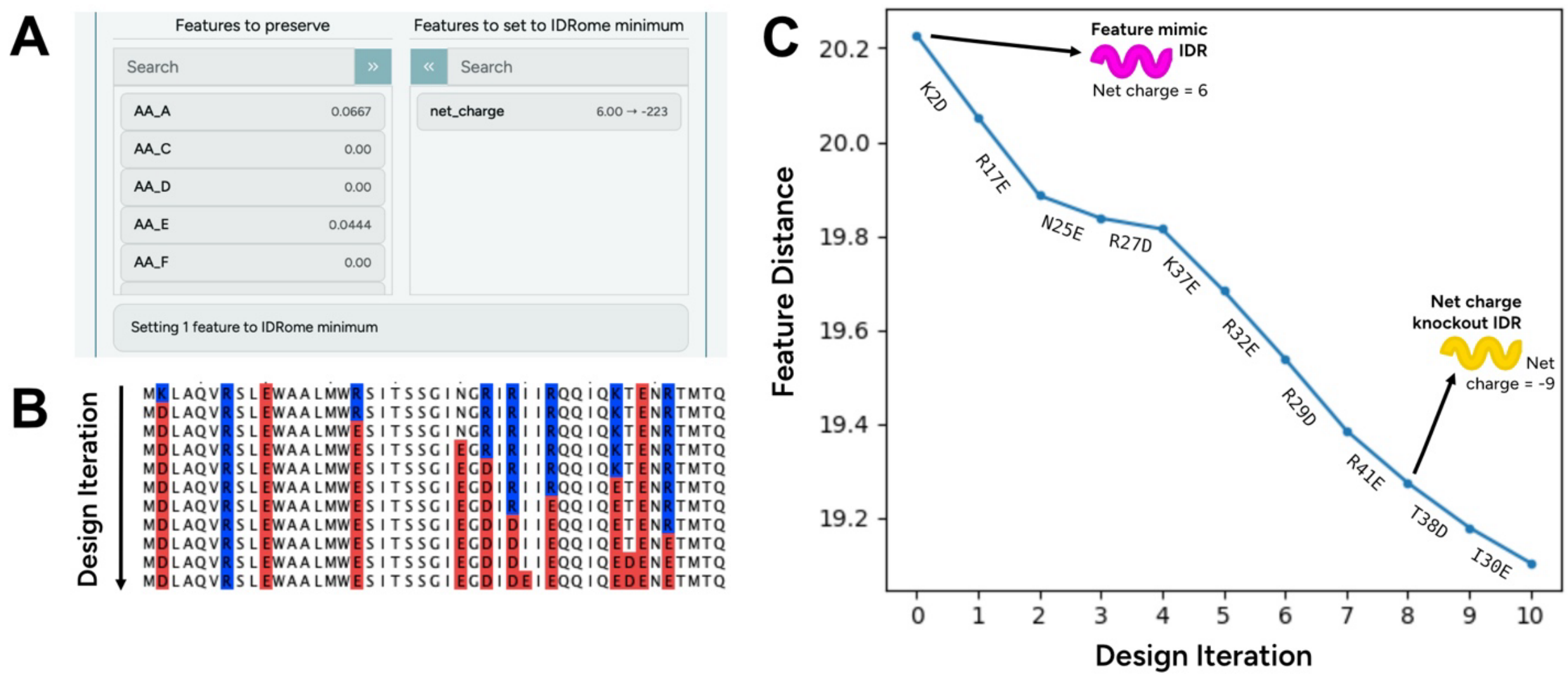

Supplementary Figure 1 – Cox15 IDR net charge knockout

(A) FAIDR-Desi web page showing attempt to knock out the net charge of Cox15 IDR (residues 1-45), by setting its net charge to the IDRome minimum. (B) Sequences generated in the first ten design iterations. Residues considered by our program to be positively charged and negatively charged are highlighted in blue and red respectively. (C) Feature distance and mutations path of first ten design iterations, with experimentally tested constructs labelled.

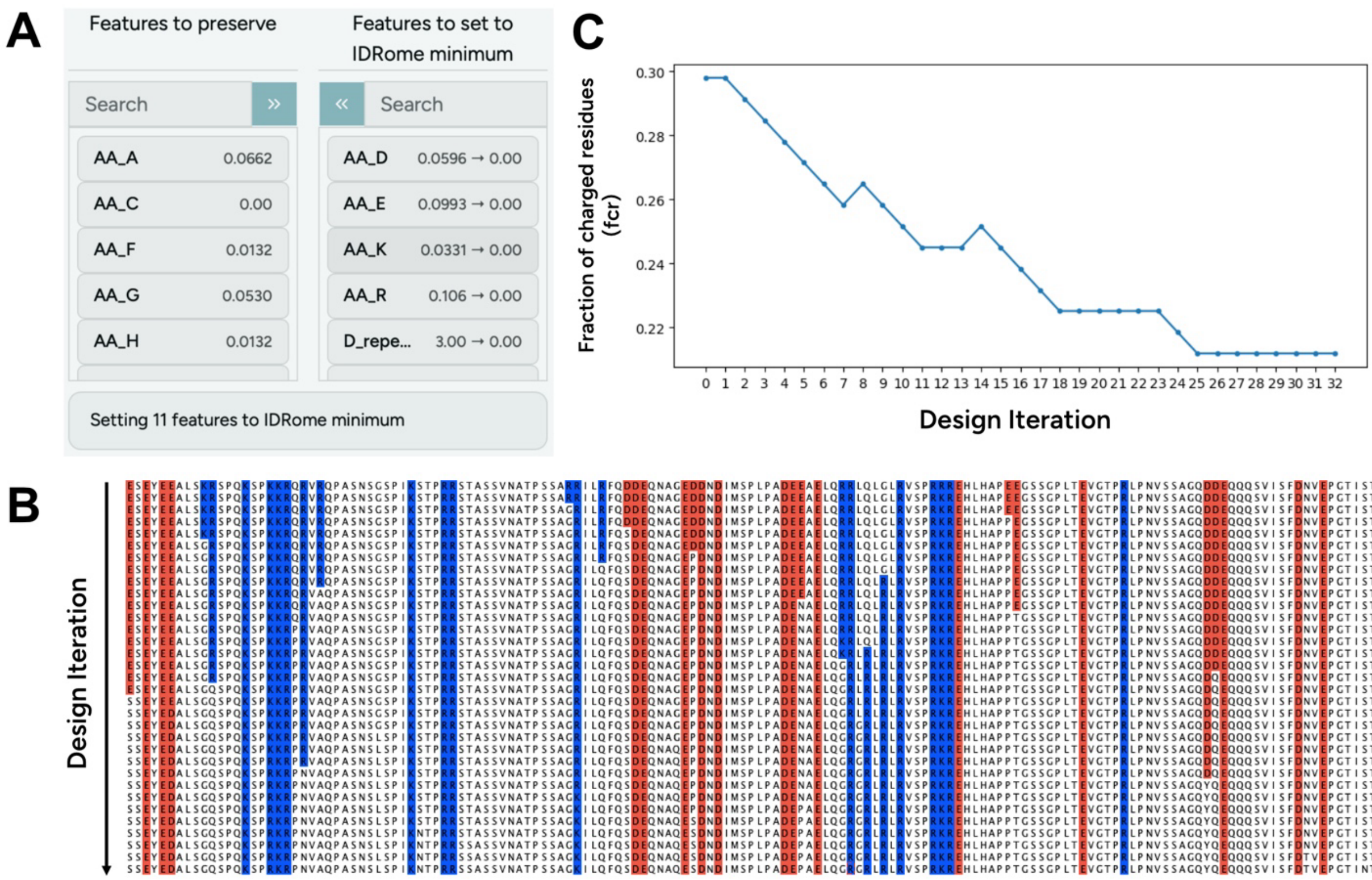

Supplementary Figure 2 – Unsuccessful Mcm3 IDR charged residue knockout

(A) FAIDR-Desi web page showing attempt to knock out the net charge of Mcm3 IDR (residues 750-900) by zeroing 11 features related to charged residues. (B) Sequences generated by the design algorithm. Residues considered by our program to be positively charged and negatively charged are highlighted in blue and red respectively. (C) The fraction of charged residues for each design iteration.
